# Search for Biomarkers for the LC-ESI-QqQ Determination of Phenoxymethylpenicillin Treatment in Raw or Cooked Chicken Meat Samples

**DOI:** 10.1021/acs.jafc.4c02060

**Published:** 2024-05-29

**Authors:** Javier Giménez-López, Jéssica Jiménez-Murcia, Alexandra Junza, Cristina Minguillón, Dolores Barrón

**Affiliations:** † Departament de Nutrició, Ciències de l’Alimentació i Gastronomia, Campus de l’Alimentació de Torribera, Universitat de Barcelona, Avda. Prat de la Riba 171, Sta Coloma de Gramenet, 08921 Barcelona, Spain; ‡ Department Enginyeria Química i Química Analítica, Universitat de Barcelona, Martí i Franquès, 1-11, 08028 Barcelona, Spain; § Institut de Recerca en Nutrició i Seguretat Alimentaria, Universitat de Barcelona, (INSA-UB), Institut de Recerca en Nutrició i Seguretat Alimentaria, Universitat de Barcelona (INSA-UB, Recognized as a Maria de Maeztu Unit of Excellence Grant (CEX2021-001234-M)), 08007 Barcelona, Spain

**Keywords:** phenoxymethylpenicillin, PENV, cooking, phenoxymethylpenicillin metabolites, chicken meat, grilling, boiling

## Abstract

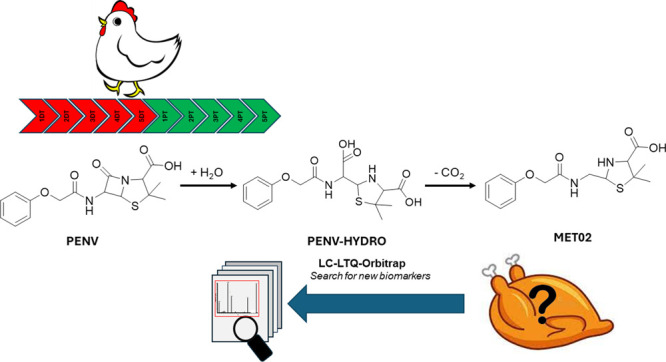

The high standards required for food safety make it necessary to trace unambiguously raw or cooked food products coming from medicated animals. Nevertheless, considering the lability of β-lactams and their degradation, the detection of the presence of antibiotics in meat either raw or submitted to a cooking process is not easily affordable. To achieve this goal, an evaluation of the effect of common domestic cooking procedures, such as boiling and grilling, on the fate of phenoxymethylpenicillin (PENV) residues was performed. Finally, in this work, the penilloic acid from PENV (**MET02)** and the corresponding penicilloic acid (**PENV-HYDRO)** are suggested as biomarkers. These compounds present the highest relative abundances 5 days after the treatment was stopped (**5PT**) and show enough thermal stability to be considered suitable biomarker candidates for the pharmacological treatment instead of the parent compound. Nevertheless, the peaks corresponding to **MET02** are significantly more intense than those for **PENV-HYDRO**, which makes preferential the use of **MET02** to perform the control of samples.

## 1 Introduction

Antibiotics are used in human and animal medicine to treat infections by eliminating or preventing the growth of microorganisms. Among the different types of antibiotics, those belonging to the β-lactam group have been widely used for this purpose. The β-lactam antibiotics, whose characteristic structural feature is the presence of the four-membered β-lactam ring, can be further classified into several groups. Among them, penicillins, cephalosporins, and carbapenems are the most frequently used.^1^

The extensive or inappropriate use of these drugs in animals may cause their accumulation in different tissues, such as muscle or the liver. The low concentration of these residues in food from animal origin devoted to human consumption makes possible toxic effects unlikely. However, allergy reactions in sensitive individuals cannot be discarded.^2^ Moreover, the extensive illicit use of antibiotics may foster the appearance of antibiotic-resistant bacteria and constitutes a potential threat to human health.^3^ In this context, some studies have been devoted to the evaluation of the toxic effects of benzylpenicilloic acid or benzylpenicillin (PENG).^4,5^

To protect humans from uncontrolled exposure to any veterinary drugs, a withdrawal time has been imposed. The withdrawal time has been defined as the interval of time between the administration of a drug to the animal until its slaughter. This period ensures a content of drug residues in meat below a maximum residue limit (MRL).^2^ MRL values for drugs, including some β-lactam antibiotics, have been established by the European Commission in animal products, such as milk and edible tissues.^6^ For β-lactam antibiotics, the MRLs in tissues range from 25 to 300 μg/kg for penicillins, with 25 μg/kg being the MRL for phenoxymethylpenicillin (PENV). This low MRL involves the need for analytical methods with high sensitivity and specificity to determine β-lactams in foodstuffs. Different methods have been developed to analyze residues of β-lactams in several matrices.^7−13^ Only a few studies are focused on the analysis of metabolites or transformation products of β-lactam antibiotics.^7,9,14,15^ However, to the best of our knowledge, none of the studies focus on compounds derived from PENV.

Many studies highlight the heat instability of β-lactam antibiotics in animal-derived products.^5,16,17^ It is described that some penicillins, such as PENG, ampicillin (AMPI), and amoxicillin (AMOX), have a degradation path favored by heating the matrix where it is located. At first, a hydrolysis takes place, resulting in the β-lactam ring opening. Subsequently, the generated penicilloic acid loses the carboxyl group that originates the corresponding penilloic acid.^5,9,18,19^ Penillic, penicilloic, and penilloic acids are compounds formed in penicillin-spiked milk and yoghurt after heat treatment and fermentation.^20^ Some authors described a decrease in the β-lactam antibiotic content in animal food products after thermal treatments.^21,22^ In this sense, cooking food results in the degradation of the drug and the formation of different compounds derived from the administered drug.^2,21,23−25^ Among the cooking treatments applied, boiling and grilling are the most common. Boiling is a simple cooking procedure for meat and involves the treatment of the matrix in water at 100 °C. Grilling allows consumers to prepare a quick meal using a direct heat source exposing food to a temperature up to 260 °C. The resulting grilled meat shows a characteristic aroma like that achieved by roasting.^25^

At present, there is a need to trace unambiguously cooked or raw food products coming from medicated animals. Nevertheless, considering the lability of β-lactams and their degradation, the detection of the presence of antibiotics in meat submitted to a cooking process is not easily affordable. In this context, the aim of this work is to identify compounds derived from PENV as more accurate markers for the presence of β-lactams than our own antibiotic. To achieve this goal, a previous evaluation of the effect of common domestic cooking procedures, such as boiling and grilling, on the fate of PENV residues is required. The study will focus on samples of the liver and muscle of chicken previously medicated with PENV.

## 2 Experimental Procedures

### 2.1 Chemicals and Reagents

**Figure 1:**
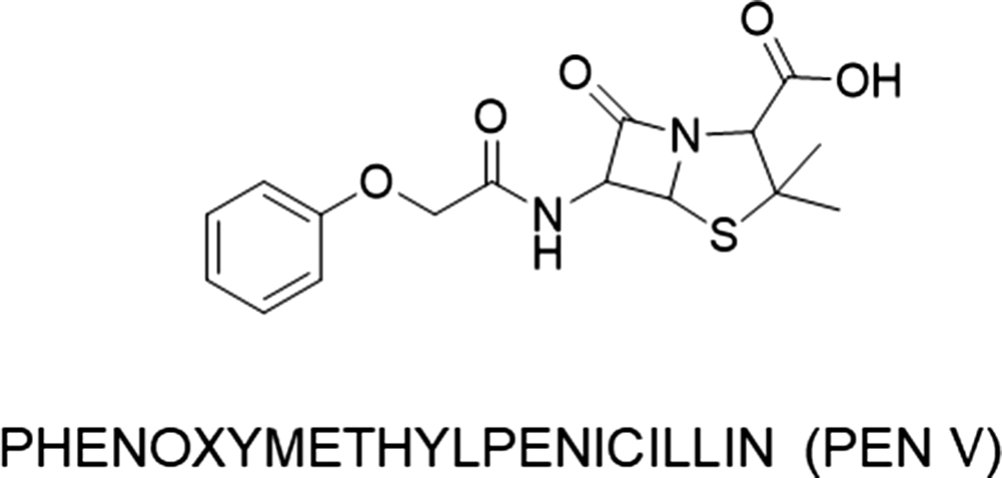
Structure of PENV.

The following reagents and solvents were used during the sample treatment process. Glacial acetic acid (HAcO), formic acid (HCOOH), hydrochloric acid (HCl) 37% v/v, and ammonium hydroxide 25% v/v were purchased from Scharlau (Barcelona, Spain); acetonitrile (MeCN), methanol (MeOH), sodium hydroxide (NaOH), ammonium chloride (NH_4_Cl), and potassium dihydrogen phosphate (KH_2_PO_4_) were obtained from Panreac (Barcelona, Spain). Anhydrous citric acid (HCit) was obtained from Sigma-Aldrich (Steinheim, Germany). In addition, anhydrous magnesium sulfate (MgSO_4_) was obtained from Sigma-Aldrich (St. Louis, MO, USA), end-capped octadecyl silica gel (C18) from Agilent Technologies (Santa Clara, CA, USA), and primary-secondary amine (PSA) 40–60 μm was purchased from Scharlau Sharlab (Barcelona, Spain). All reagents were of analytical grade, unless otherwise indicated. The standard PENV (Figure 1) was purchased from the European Pharmacopeia (Strasburg, France).

Extraction cartridges OASIS HLB 3 cm^3^ (60 mg), supplied by Waters (Milford, MA, USA), were used in the SPE procedure. In addition, centrifugal filter units Ultrafree-MC-GV Durapore-PVDF 0.22 μm from Merck Millipore were used to filter samples before injection into the LC-MS/MS system.

Ultrapure water was generated by a LaboStar Milli-Q purification system from Evoqua Water Technologies (Pittsburgh, PA, USA).

### 2.2 LC-MS/MS Instrumentation and Conditions

Liquid chromatography separation was carried out on a Symmetry C8 column (50 × 2.1 mm, 5 μm) obtained from Waters (Milford, MA, USA). The flow rate was adjusted to 0.3 mL/min and the injection volume was 10 μL. The mobile phase consisted of a binary solvent system: solvent A, water with 0.1% HCOOH, and solvent B, MeCN with 0.1% HCOOH. A gradient elution was programmed as follows: initially, B was maintained at 4% for 2 min, from 2 to 4 min B increased to 20%, from 4 to 6 min B increased again to 50%, and from 6 to 8 min B is maintained at 50%. Finally, B decreased to 4% in 1 min and was maintained at this percentage for 3 min to recover initial conditions.

An Acquity-Ultra Performance LC system equipped with a thermostatic autosampler from Waters (Milford, MA, USA) and coupled to an API 3000 triple-quadrupole mass spectrometer from PE Sciex (Framingham, MA, USA), using a turbo ion spray source in positive mode, was used in the quantification of PENV in samples. Multiple reaction monitoring (MRM) and positive ionization mode with a dwell time of 200 ms were performed. LC–MS/MS conditions were optimized by direct injection of a standard solution of PENV at a concentration of 1 mg·L^–1^. The following parameters were optimized: capillary voltage 4500 V, nebulizer gas (N_2_) 10 (arbitrary units), curtain gas (N_2_) 12 (arbitrary units), collision gas (N_2_) 15 (arbitrary units), declustering potential (DP) 40 V, focusing potential 175 V, and entrance potential 5 V. Drying gas (N_2_) was heated to 400 °C and introduced at a flow rate of 4500 mL·min^–1^. MS/MS product ions were produced by collision-activated dissociation (CAD) of the selected precursor ion. Two transitions were followed. The most intense transition (351 → 160 (15 V)) was used for quantification of PENV, while the second (351 → 114 (45 V)) ensures identification. The system was controlled by using Analyst 1.4.2 software from Applied Biosystems (Foster City, CA, USA).

An LC-LTQ-Orbitrap system was used for the characterization of metabolites. This system consisted of an Accela LC system equipped with a thermostatic autosampler and coupled to an LTQ Orbitrap Velos mass spectrometer, both from Thermo Scientific (Hemel Hempstead, UK). Mass spectrometry analyses were carried out on full-scan and product ion scan MS/MS modes with a mass range of 150–550 Da. The resolving power was 30,000 for full-scan mode and 15,000 for MS/MS events. Positive ionization mode was used in all experiments. A source voltage of 3500 V and a capillary temperature of 300 °C were applied. Collision energy (CE) in CID mode (10–80 V) was used for the MS/MS experiments. The *m*/*z* 351.1009 (CE: 20 V) peak was followed in the fragmentation of PENV. The instrument was controlled using XCalibur 2.2 software from Thermo Fisher Scientific (Hemel Hempstead, UK).

### 2.3 Auxiliary Equipment

An analytical balance AM1000 (±0.0001 g) from Mettler Toledo (Greifensee, Switzerland) and a technical balance 440–45N (±0.1 g) from Kern Pharma (Barcelona, Spain) were used in the weighing of reagents and samples. A potentiometer micro-pH 2002 (±0.1 mV) and a combined pH electrode 5203 both from Crison (Barcelona, Spain) were used in the preparation of the buffer solutions.

A vortex mixer VX-200 from Labnet International (Edison, NJ, USA), an ultra sonicator from J.P. Selecta (Barcelona, Spain), a centrifuge MIKRO 220R from Hettich Zentrifuguen (Lauenau, Germany), and an evaporator MiVac Quattro concentrator Duo Pump with SpeedTrap from GeneVac (Warminster, PA, USA) were used during sample treatment. The SPE procedure was performed on a vacuum manifold with disposable liners for 24 cartridges connected to a vacuum tank from Supelco (Bellefonte, PA, USA).

### 2.4 Preparation of Standard and Stock Solutions

A stock solution of PENV in water at a concentration of 250 mg·L^–1^ was prepared. Several solutions of PENV at various concentrations, according to their later application, were prepared. Thus, a solution at 5 mg·L^–1^ was used in the preparation of fortified samples at 300 μg·L^–1^ involved in the experimental design. Solutions at 0.5 and 10 mg·L^–1^ were used to establish the calibration curve. A solution at 1 mg·L^–1^ was prepared to evaluate the effect of pH on stability, and solutions at 25 and 200 μg·kg^–1^ in tissues were prepared to evaluate the reproducibility of the analytical method.

Buffer solutions used in the experimental design of the QuEChERS procedure consisted of an aqueous HCit solution (0.1 M) adjusted at pH 4.0, 4.5, 5.5, 6.0, 6.5, and 7.0, with a solution of NaOH (2 M).

Regarding the SPE procedure for the aqueous phase resulting from boiled samples, buffer solutions of potassium dihydrogen phosphate (0.1 M), adjusted at pH 2.0 and 3.0 with a solution HCl (0.3 M), or adjusted at pH 7.5, 8.0, and 8.5 adding a solution of NaOH (2 M), were prepared. A buffer solution of NH_4_Cl (0.1 M), adjusted at pH 10.0 with a solution of NaOH (2 M), was also used.

### 2.5 Origin of Samples and Animal Pharmacological Treatment

Chicken tissues from nonmedicated animals were used in both blank samples and in the preparation of the calibration curve. Chicken tissues from medicated animals resulted from animals subjected to the following therapeutic treatment. Thus, chickens were submitted to a dose of 20 mg·kg^–1^ of PENV dissolved in drinking water for 5 days. The treatment water was changed every 12 h.

Two specimens (A1 and A2) were slaughtered on the third day of the pharmacological treatment (during treatment **3DT** samples). Two chickens (A3 and A4) were slaughtered on the fifth day of the pharmacological treatment (during treatment **5DT** samples). Additional chickens were slaughtered 3 days (A5 and A6, posttreatment **3PT** samples) or 5 days (A7 and A8, posttreatment **5PT** samples) after stopping the pharmacological treatment. Two nonmedicated specimens (A9 and A10, nonmedicated **NM** samples) randomly selected were also slaughtered. All animals were handled and sacrificed according to ethical protocols. Liver and muscle tissue samples from all different chickens were stored at −20 °C until sample treatment.

### 2.6 Sample Preparation for PENV Stability Study

The stability of PENV to pH and contact time (**CT**) with tissue was tested. To evaluate the effect of pH on the stability of PENV, a working solution of 1 mg·L^–1^ was left in contact with buffers at several pH values (2.0–8.0) and kept at −20 °C until analysis. To evaluate the effect of **CT** of PENV with tissues on the stability of this β-lactam, an amount of 2 g (±0.1 mg) of minced chicken muscle or 1 g (±0.1 mg) of minced chicken liver was introduced into a 50 mL capped polypropylene centrifuge tube. A solution of PENV at 5 mg·L^–1^ was added until a final concentration of 300 μg·kg^–1^ was reached in each sample. Samples were sonicated in an ultrasonic bath for 2 min and left in contact with the antibiotic for 1 h (**1CT**) or 24 h (**24CT**) at 4 °C. PENV was extracted from samples using the QuECHERs method (Section 2.9.1).

Samples were analyzed in triplicate using an LC-LTQ-Orbitrap apparatus (Section 2.2). Results were compared to those obtained for blank samples.

### 2.7 Cooking Procedure

Samples, consisting of 2 g (±0.1 mg) of muscle or 1 g (±0.1 mg) of liver conformed as a “hamburger” of approximately 1 cm in diameter, were exposed to two different cooking treatments: boiling (**B**) and grilling (**G**). These tissues were also analyzed before any cooking treatment (raw meat, **R**). The grilling procedure consisted of a 2 min/side in both muscle and liver samples. The boiling procedure consisted of a 5 min treatment in 10 mL of Milli-Q water. The mixture was filtered to separate the boiled sample (**B**) from the boiling water (**BW**). The two parts were analyzed separately.

### 2.8 Optimization of the QuEChERS Method

Sample treatment and cleanup by SPE were already optimized in our laboratory on the occasion of precedent studies.^26,27^ However, this is not the case for the QuEChERS procedure. To optimize the QuEChERS extraction method, a Plackett–Burman design was applied to screen the significant experimental factors in the procedure. Subsequently, the Doehlert design was used to find the optimum conditions for selected factors. Blank chicken tissues (muscle and liver) were used for these purposes.

In the Plackett–Burman design, five factors were considered. Each factor was studied at two levels (low and high): MgSO_4_ (0 and 1000 mg), C18 (0 and 600 mg), PSA (0 and 600 mg), shaking time (20 and 60 s), and pH (3 and 10). Considering all possible combinations, 12 independent runs were performed for each tissue (24 in total).

In the Doehlert design, the most influential factors detected previously were studied at additional levels to obtain the maximum information and a better prediction. The considered factors for the muscle matrix were: 7 MgSO_4_ amounts (0, 167, 334, 500, 667, 834, and 1000 mg), 7 PSA quantities (0, 100, 200, 300, 400, 500, and 600 mg), 5 pH levels (3, 4.5, 6.5, 8.5, and 10), and 3 C18 amounts (0, 300, and 600 mg). The considered factors for the liver matrix were: 7 MgSO_4_ amounts (0, 167, 334, 500, 667, 834, and 1000 mg), 7 pH levels (3.0, 4.5, 5.5, 6.5, 7.5, 8.5, and 10.0), 5 PSA amounts (0, 150, 300, 450, and 600 mg), and 3 C18 quantities (0, 300, and 600 mg). For each matrix, 23 experiments were performed (46 experiments in total), and 3 of them were replicates of the central point.

### 2.9 Sample Treatment and Cleanup

The QuEChERS procedure was used for solid samples (**R**, **G**, **B**), whereas the SPE method was used in the cleanup and preconcentration of **BW** samples.

#### 2.9.1 QuEChERS

An amount of 2 g (±0.1 mg) of minced chicken muscle or 1 g (±0.1 mg) of minced chicken liver was introduced into a 50 mL capped polypropylene centrifuge tube. A mixture of 8 mL of MeCN and 2 mL of HCit buffer solution (pH 3.0 for the muscle tissue and pH 7.0 for the liver tissue) was added. Then, the tubes were sonicated (5 min) and centrifuged at 10,000 rpm at 10 °C (5 min). The supernatant was transferred into a 15 mL capped polypropylene centrifuge tube with the optimized amount of sorbents (MgSO_4_ and PSA). Concretely, 700 mg of MgSO_4_ and 600 mg PSA were used for muscle matrices, while 1000 mg of MgSO_4_ and 600 mg PSA were used for liver matrices.

After shaking the QuEChERS tube for 30 s, the mixture was centrifuged at 5000 rpm and 10 °C (5 min). Finally, a 5 mL aliquot of the supernatant was completely evaporated and the mixture was reconstituted with 200 μL of water. The mixture was vortexed for 30 s and filtered (0.22°μm) by centrifugation at 10,000 rpm (5 min). Finally, the filtered solution was transferred to LC-MS/MS vials and kept frozen (−20 °C) until analysis. All experiments were performed in triplicate.

#### 2.9.2 SPE

The **BW** samples were cleaned using SPE Oasis HLB cartridges. Initially, 1 mL of MeOH, 1 mL of water, and 1 mL of KH_2_PO_4_ buffer solution (pH 10) were passed through the cartridge to condition the sorbent. Next, 5 mL **BW** sample followed by 3 mL water were passed through the cartridge.^26,27^ Finally, analytes were eluted by adding 5 mL of MeOH to the cartridge. The obtained extract was reconstituted as described above for samples submitted to the QuEChERS procedure and kept frozen (−20 °C) until analysis. All samples were analyzed in triplicate.

### 2.10 Quality Parameters

To evaluate the linearity of the analytical procedures applied, calibration curves were prepared. For each type of tissue, muscle or liver, calibration curves were obtained for **R** and **BW** samples. The blank **BW** sample, required to obtain the calibration curve, was prepared by boiling blank tissue under the same conditions as real samples. The curves were obtained at 7 levels of concentration within the range of 5–300 μg·kg^–1^ for the liver, while for muscle 6 levels of concentration within the range of 2.5–200 μg·kg^–1^ were used. Also, each concentration level was prepared and assayed twice.

The precision of the QuEChERS procedure was evaluated in terms of repeatability (intraday precision). Blank chicken muscle **R** was used to perform the study. The samples were prepared the same way as the samples for the calibration curve. The samples were spiked at 2 different concentration levels of 25 and 200 μg·kg^–1^. To evaluate the intraday precision, 10 replicates at each of these levels were prepared.

The LOQ was evaluated in muscle and liver tissues using the calibration line method, considering a signal-to-noise ratio of 10.

### 2.11 Quantification of PENV

Calibration curves were constructed within the range of 2.5–200 μg·kg^–1^ for muscle and 5–300 μg·kg^–1^ for the liver. The tissue, either muscle or liver, and water samples were quantified using LC-ESI-QqQ and the appropriate calibration curve.

The concentration of PENV was determined in chicken muscle and liver tissues for the **R** samples and the three cooking conditions considered (**G**, **B**, and **BW**).

### 2.12 Data Treatment

The LC-ESI-QqQ data were processed by using Analyst 1.6.2 software from Applied Biosystems (Framingham, MA, USA). This software provides the chromatograms obtained in targeted analysis. The peak areas belonging to PENV were integrated for quantification.

The LC-LTQ-Orbitrap data were processed with Compound discoverer 2.1 software from Thermo Fisher Scientific (Waltham, MA, USA). This software permits one to perform a targeted analysis for specific compounds in many samples simultaneously. As a result, samples containing the considered compound are easily located.

Data obtained from blank samples were taken as a reference when the effect of contact time of the antibiotic with the matrices was studied. In parallel, data from cooked samples were compared with the raw ones when the effect of the cooking process on the evolution of metabolites and transformation products (**TP**s) was considered.

Results were filtered considering the following restrictions: working time range (*t*_R_ 2–8 min), tolerance in the retention times of the pics for the same *m*/*z* (0.2 min), mass range for PENV (*m*/*z* 150–550), peak area higher than 5000, and a mass defect filter (0.02 < MDF < 0.17). The application of such filters permits to decrease in the probability of ion artifacts. Additional constraints, such as the presence of ions in replicates and not in blank samples, and an area over 10% that of the parent ion for the considered ion, were also applied.

## 3 Results and Discussion

An optimized and validated analytical method (extraction and quantification) for PENV in the different matrices is needed when evaluation of the effect of the cooking treatments on this β-lactam antibiotic is undertaken. QuEChERS and SPE extraction methods in both muscle and liver chicken tissues were used.

### 3.1 QuEChERS Procedure Optimization

In the optimization of the QuEChERS extraction procedure of PENV, the amounts of PSA, C18, and MgSO_4_ in the d-SPE mixture used, as well as the stirring time and pH, were considered. To evaluate the effect of these five factors on the extraction, a Plackett–Burman design was applied (Section 2.8). The samples were analyzed by LC-ESI-QqQ using the signal corresponding to the quantification transition for PENV (351 → 160). The identification transition (351 → 114) was used for confirmation. From the Pareto diagrams, it was concluded that stirring time does not affect the results. Consequently, this parameter was discarded from the optimization design. The influence of the four remaining variables was, from the most to the least influent factor, the amounts of MgSO_4_ and PSA, pH, and C18 in muscle. The amount of MgSO_4_ was also the most influent factor in liver matrices followed by pH and the amounts of PSA and C18.

Subsequently, a Doehlert design^28^ was carried out to quantitatively evaluate the optimal conditions of the four factors studied. In the Doehlert design, the factors are applied at a different number of levels (3, 5, or 7) depending on their significance. Different designs were performed for muscle and liver tissues considering the difference in significance of the four factors when treating the two matrices.

The number of levels considered for the amounts of MgSO_4_ and PSA was 7, covering the ranges 0–1000 and 0–600 mg, respectively, for muscle matrices. Analogously, 5 levels were considered for pH (3–10) and only 3 levels for the amount of C18 (0–600 mg).

When dealing with liver matrices, 7 levels were considered for the amount of MgSO_4_ (0–1000 mg) and pH (3–10) and 3 levels for the amounts of PSA (0–600 mg) and C18 (0–600 mg). For muscle matrices, 700 mg of MgSO_4_, 600 mg of PSA, and pH 3.0 were determined to be the optimum values for PENV extraction. For liver matrices, 1000 mg MgSO_4_, pH 7.0, and 600 mg PSA were considered to be the optimum conditions. In the two cases, the best results were obtained in the absence of C18.

### 3.2 Quantification of PENV

The linearity, the precision, and the LOQ of the method used to quantify PENV were established. The linearity was established for the two tissues used in the study, namely, muscle and liver. The calibration curves were prepared considering the ranges 2.5–200 μg·kg^–1^ for muscle and 5–300 μg·kg^–1^ for liver, using muscle and liver blank tissues. Calibration curves for **BW** of tissues were also prepared. All samples (**R**, **G**, **B, and BW**) of muscle and liver matrices were quantified using the appropriate calibration curve by LC-ESI-QqQ. Correlation coefficients (R) greater than 0.992 were obtained in all instances.

Additionally, precision was also established at two levels (25 and 200 μg·kg^–1^). The QuEChERS method has proven to be reproducible given that RSD values at 25 μg·kg^–1^ were 10% for muscle and 11% for liver. The interday precision was 9% either for muscle or liver. In all cases, results fall within the acceptance criteria for the validation of analytical methods, which is RSD ≤15%.^29^

The LOQ values for a ratio S/N of 10, obtained in muscle and liver matrices, were 2.5 and 5 μg·kg^–1^, respectively. These values are below the MRL values established in European regulations.

The determination of PENV concentrations was carried out by LC-ESI-QqQ following the method described (Section 2.9, Section 2.10, and Section 2.11). Three independent replicates were analyzed for each animal and tissue. The results of the PENV quantification are shown in Table A (Supplementary Data). The values obtained for the two tissues were compared for each cooking treatment, **R**, **G**, **B,** and **BW**.

Samples from specimens A9 and A10 come from **NM** animals and are used as blank tissue. PENV resulted to be detectable and quantifiable only for **R** samples coming from animals medicated and slaughtered immediately after 3 or 5 days of treatment (**3DT** and **5DT**). In the case of liver samples, this is so only for one of the specimens studied, A1 (**3DT**) and A4 (**5DT**). When comparison is possible (A1 and A4), the concentration of PENV resulted to be higher in the liver than in muscle, which points to an accumulation of the drug in this organ.

When muscle samples are submitted to either grilling or boiling, the level of PENV decreases down the LOQ, being undetectable in **BW** samples. Regarding liver samples, PENV can be detected although unquantifiable after grilling or boiling, only in those coming from specimens A1 and A4. This decrease in PENV content can be attributed to the lability of PENV under the effect of the cooking treatments.

Regarding **PT** samples, in which pharmacological treatment was stopped three or 5 days before slaughtering, PENV resulted to be undetectable even in **R** samples. This can be the result of the progressive metabolism and degradation/transformation of PENV in the animals.

The quick transformation of PENV observed in tissues, which is favored by temperature, confirms the need for the search of an alternative marker to control medication with PENV in chicken meat.

### 3.3 Stability of PENV

Before the study of metabolization and/or transformation of PENV in **R** and cooked samples (**G**, **B**, and **BW**), the stability of this β-lactam under the conditions of the study has been considered. Thus, the effect of the simple contact with meat, which may affect the results in a calibration curve prepared with matrix, and pH used in analysis has been considered. Two values of contact time between drug and meat were studied: 1 h (**1CT**) and 24 h (**24CT**) (Section 2.6). Samples were extracted (Section 2.9) and analyzed by an LC-LTQ-Orbitrap. Results were compared with those obtained for blank samples, and only *m*/*z* values that were detected in samples and not in blank were considered. The number of *m*/*z* obtained was reduced by applying the filters described previously (Section 2.12). Nevertheless, the number of *m*/*z* signals in **1CT** and **24CT** conditions was still too high to be studied. Therefore, in order to diminish this number, only the ions present under both conditions have been considered. Table B (Supplementary Data) shows a list of these compounds (**TP01-TP12**).

**Figure 2:**
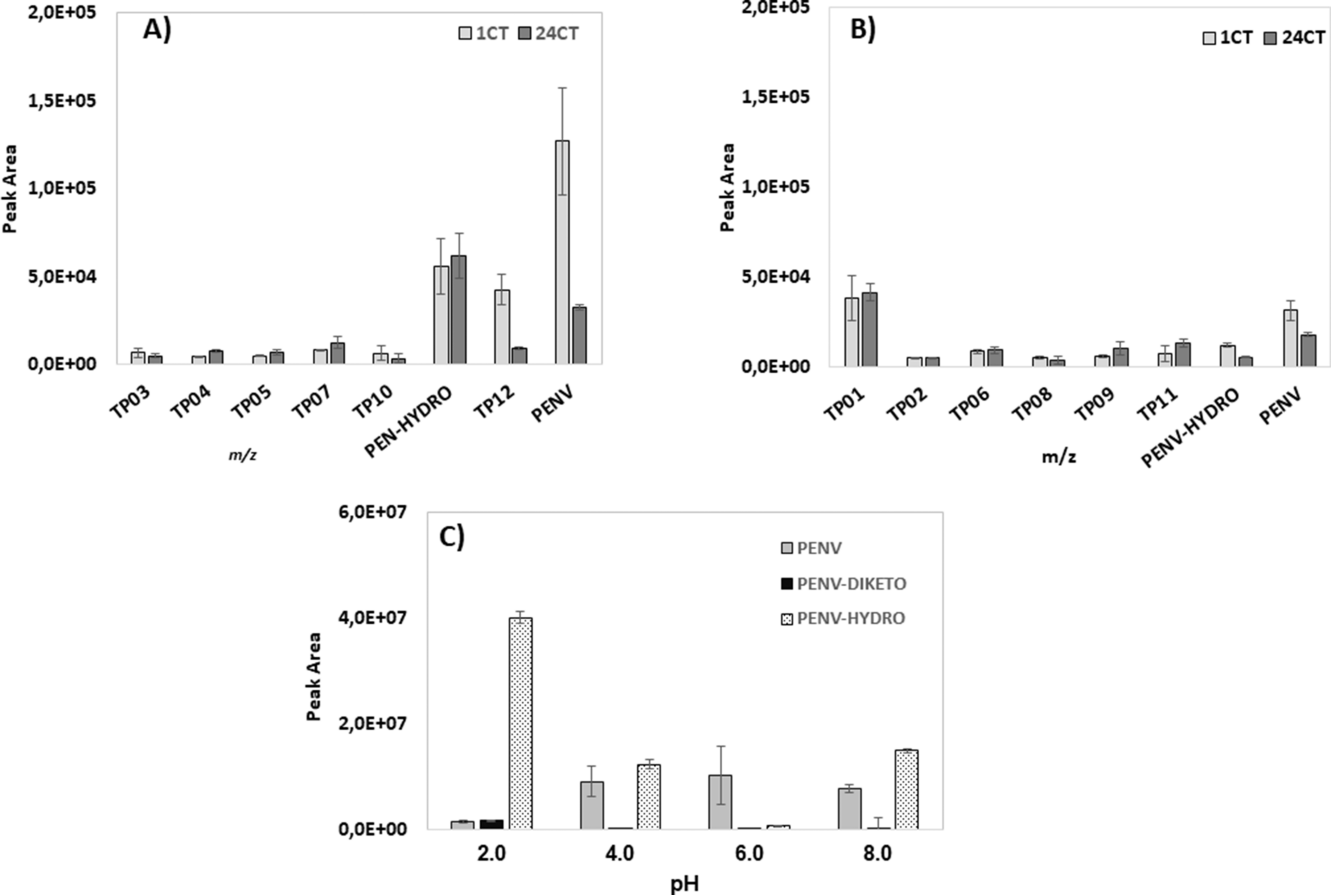
Behavior of PENV and the TP formed in contact with matrices. (A) muscle and (B) liver. 
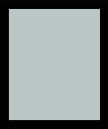
 1CT (1 h contact time); 
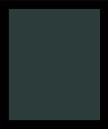
 24CT (24 h contact time). (C) Behavior of PENV with pH. PENV, 
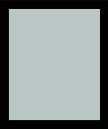
; PENV-DIKETO, 
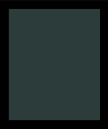
; PENV-HYDRO, 
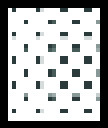
.

Figure 2 permits us to assess the significance of contact time on the presence of these compounds. Some compounds, such as **TP03** and **TP12** in muscle (Figure 2A) and **PENV-HYDRO** in the liver (Figure 2B) or PENV in the two tissues, are highly sensitive to contact time, while others are less sensitive to this parameter. Among them are **TP01**, **TP02**, **TP06,** and **TP08** in the liver and **TP10** and **PENV-HYDRO** in muscle. A third group increments their area when contact time increases (**TP04**, **TP05**, and **TP07** in muscle and **TP09 andTP11** in the liver).

Given the known lability of β-lactam antibiotics to pH modification, it is worth studying the presence of metabolites/degradation products produced by pH changes. Standard PENV was submitted to buffer solutions from pH 2.0 to 8.0 (Section 2.6). All solutions were analyzed using LC-LTQ-Orbitrap in full-scan mode (Section 2.2) and also monitoring the *m*/*z* ratio corresponding to the molecular ion of PENV. Three replicates of the solution PENV were compared with the corresponding blank solution. Thus, the *m*/*z* ions of interest are those that appear only in the samples.

A peak with the same experimental *m*/*z* as PENV (351.1014) but different retention times appears at all pH values. The structure of the diketopiperazine derivative of PENV is suggested for this compound (**PENV-DIKETO**). The formation of diketopiperazines is described for several penicillins, after the opening of the β-lactam ring.^30^ However, this is not the main degradation product produced at certain pH values.

The hydrolysis of penicillins under either acid or alkaline conditions resulting in penicilloic acid was described. Concretely, ampicillin-penicilloic acid is described to play an important role in the degradation of ampicillin.^19^ In the present case, a peak at *m*/*z* of 369.1118 (**PENV-HYDRO**) was observed at all pH values tested, being more abundant at extreme pH values. Degradation of PENV to be transformed into the diketopiperazine derivative is more important at pH 2.0 than at other pH values. However, at this pH, the **PENV-HYDRO** is the most abundant derivative (Figure 2C).

### 3.4 Effect of Cooking on Metabolite Content

Considering that meat is commonly ingested in the cooked form (**G and B**) and not in the **R** form, it seems interesting to study the effect that the cooking process may have on PENV and its metabolites or **TP**s.

Tissues from two different animals sacrificed during pharmacological treatment (**3DT** and **5DT**) and those corresponding to animals sacrificed after pharmacological treatment (**3PT** and **5PT**) were analyzed. Samples **R**, **G**, **B,** and **BW** from either muscle or liver tissues were analyzed using LC-LTQ-Orbitrap (in full-scan mode) and compared with blank tissues (samples from **NM** animals). Data were treated using the software Compound Discoverer applying the filters explained in Section 2.12. Only compounds present in samples and not in blank tissues were considered. Three independent replicates were prepared and injected for each tissue and cooking procedure.

Table 1 shows a summary of the ions found in this study presented in terms of exact mass and kind of sample (**R**, **B**, **G,** or **BW**, muscle or liver) in which the metabolite or TP is present.

**Table 1:**
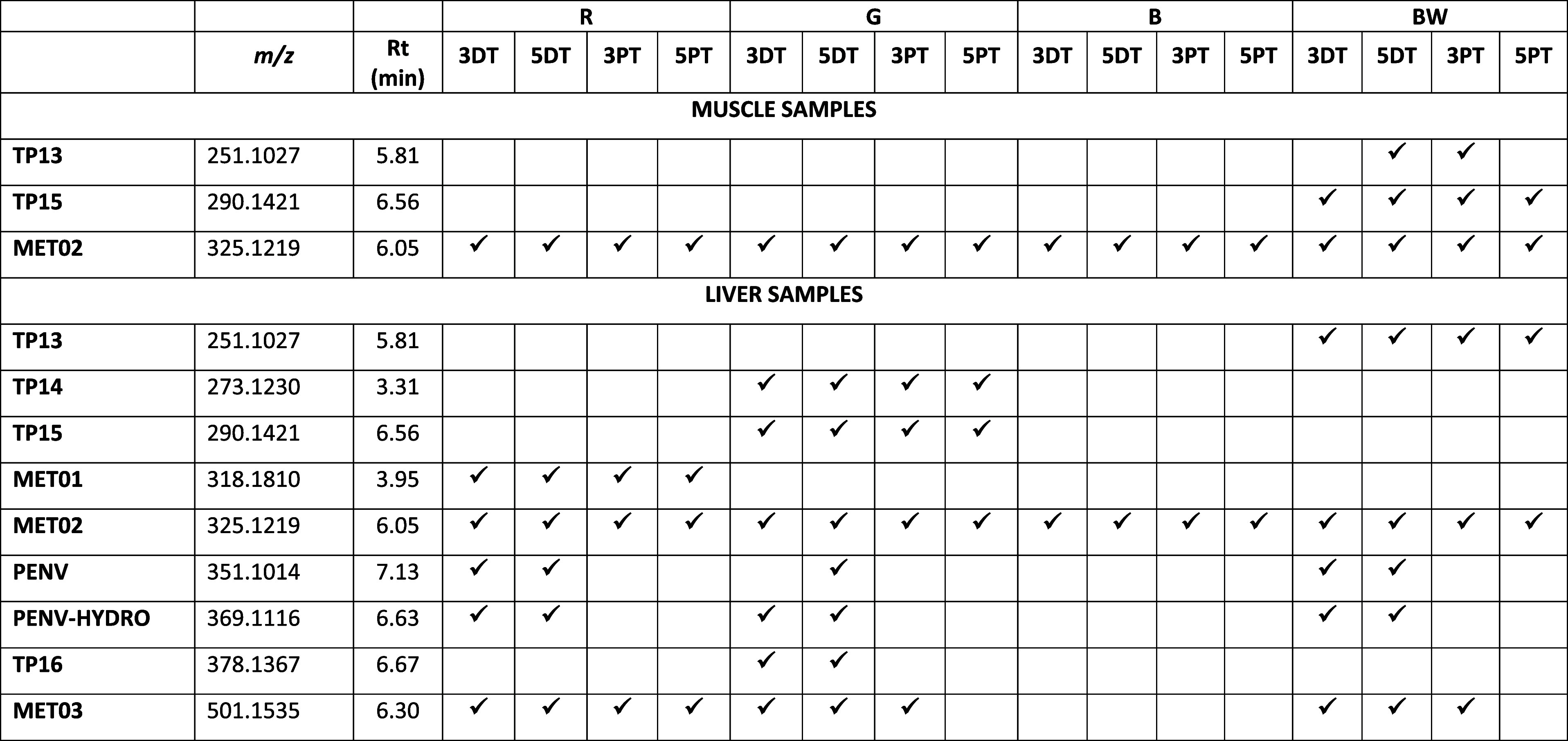
Metabolites and Transformation Products of PENV in Muscle and Liver Chicken Samples

First of all, we can find metabolites that come from transformation of the antibiotic resulting from the metabolism of the animal. These compounds can be produced without applying any thermal treatment. Therefore, they may be found in cooked samples, although they are considered metabolites only if they are present in **R** samples. These compounds were named as **MET01**, **MET02,** and **MET03**.

**Figure 3:**
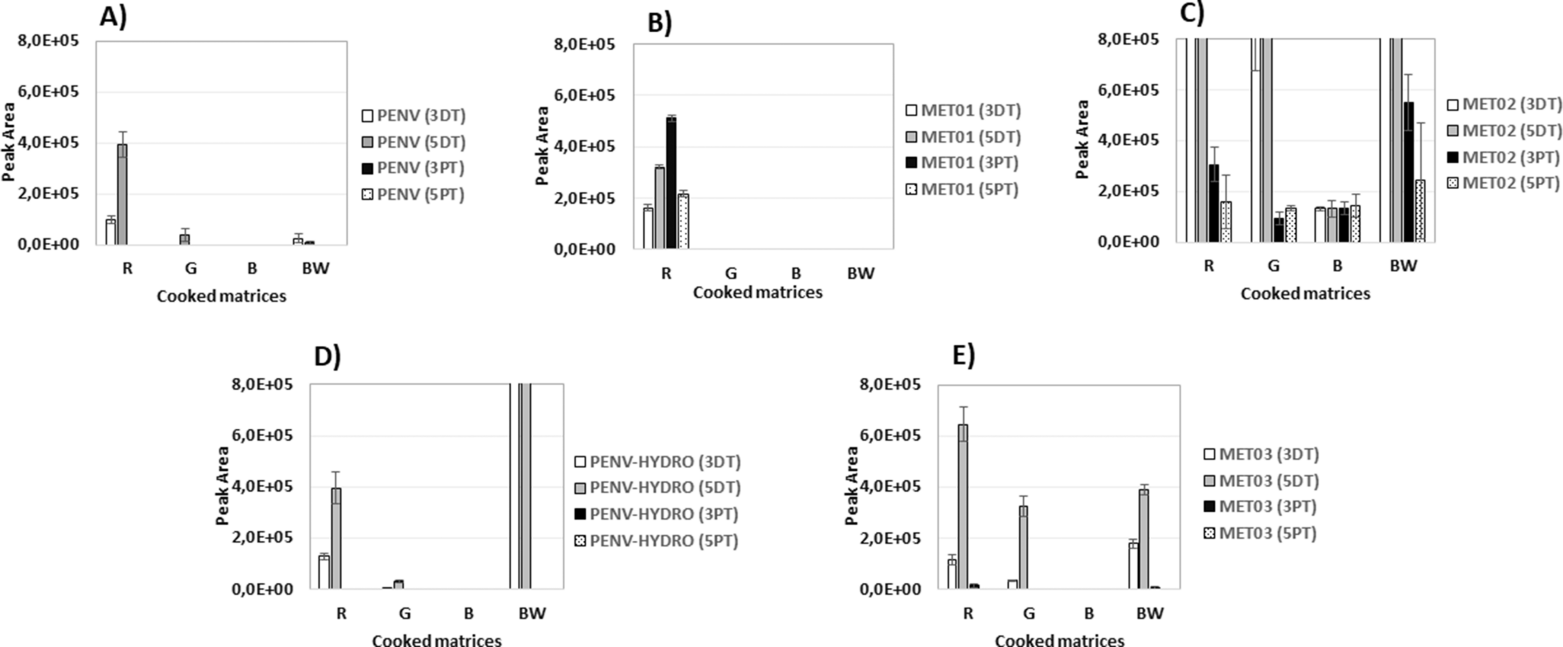
Effect of cooking procedures on PENV and metabolites during 
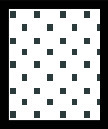
 pharmacological treatment. (A) **PENV**; (B) **MET01**; (C) **MET02**; (D) **PENV-HYDRO**; (E) **MET03**; **3DT**, 
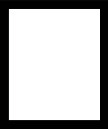
; **5DT**, 
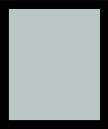
; **3PT**, 
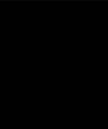
; **5PT**, 
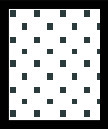
.

Figure 3 shows the behavior of these metabolites, including **PENV-HYDRO**, in liver samples when the different cooking procedures considered were applied. For comparative reasons PENV is also indicated.

**MET01** appears in the **R** samples as either **DT** or **PT**. This compound becomes undetectable when the different thermal treatments are applied, which indicates its highly thermally labile nature. A different behavior is shown by **PENV-HYDRO** and **MET03**, which remain in liver samples despite being cooked. The two compounds prove to be highly hydrophilic given that they migrate to **BW** when the sample is boiled. These two metabolites do not appear in chicken muscle. **MET02** was observed in both muscle and liver tissues, in **R** and cooked samples, and in either **DT** or **PT** samples. However, their content (between 50 to 2500 times) is higher in liver samples (Figure 3C). In contrast, PENV can only be detected in **G** and **BW** for samples obtained during treatment.

Additionally, there are some compounds which appear when a cooking procedure is applied, although they are not detectable in the **R** sample. These had been named as transformation products (**TP13**, **TP14**, **TP15,** and **TP16**) resulting from the thermal treatment (Table 1). All these compounds have been detected in the liver. Although only **TP13** and **TP15** are also present in muscle samples. Unfortunately, none of these compounds is detectable independently of the cooking procedure and time of treatment, which prevents them from being considered as an appropriate marker for medication.

### 3.5 Identification of Metabolites and Transformation Products

With the aim of identifying metabolites and **TP** formed during the cooking procedures, samples from medicated animals with PENV were analyzed using LC-LTQ-Orbitrap. Table 2 shows a summary of the ions tentatively elucidated in this study, with their molecular and structural formula, mass error, and RDB. Given the absence of commercial standards, structures were tentatively identified by the MS/MS spectrum generated from each compound and thanks to the comparison of fragments of other structurally related compounds in the METLIN database and MassFrontier software. However, in some cases this elucidation was not possible.

**Table 2:**
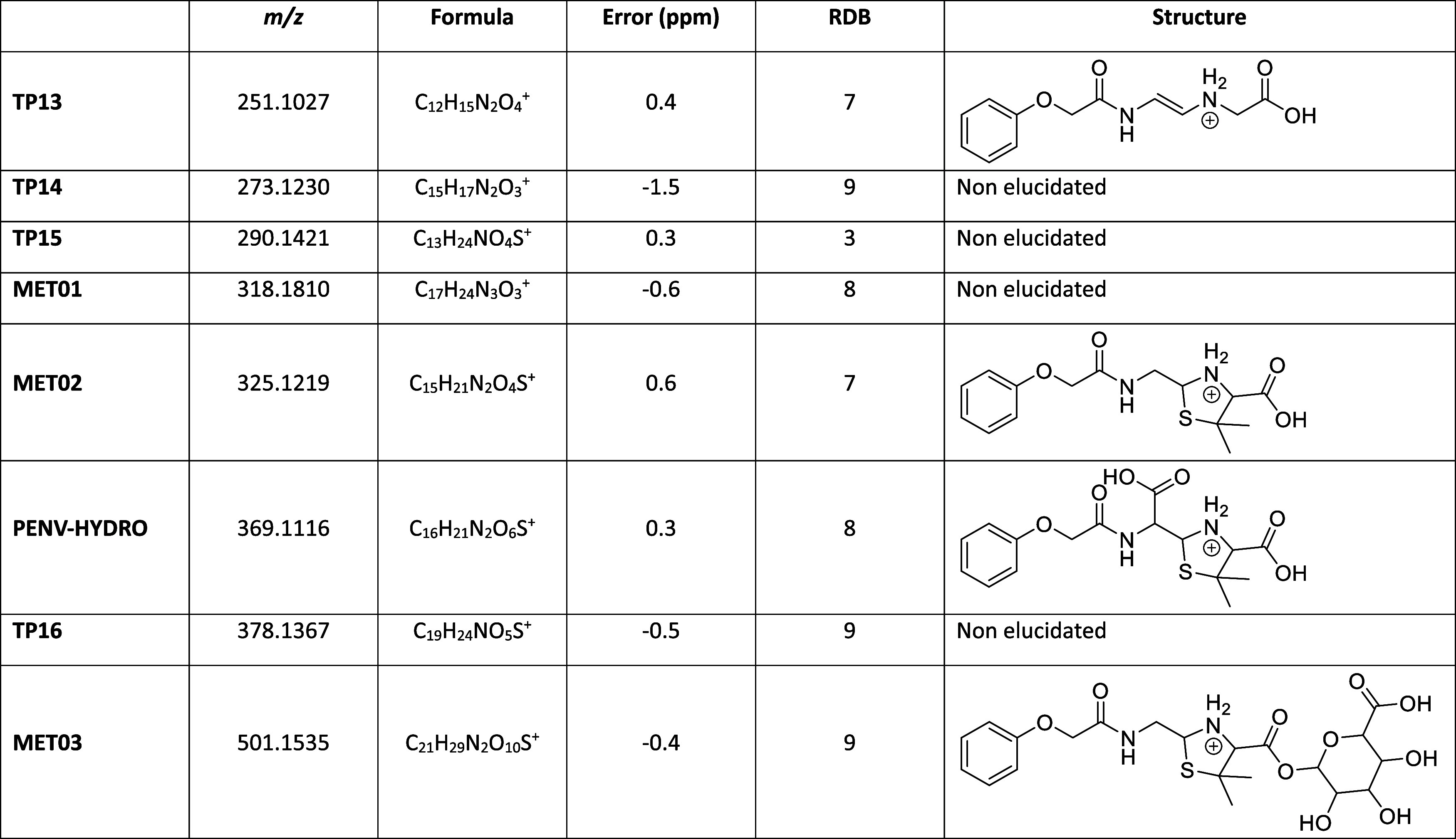
Proposed Structure of Metabolites and Transformation Products Detected in Chicken Medicated with PENV

According to the proposed structures shown in Table 2, the main biotransformation reactions of PENV affect the β-lactam ring. The known degradation caused by the acid or base of the β-lactam ring of penicillins results in **PENV-HYDRO.** The structure of **MET02** corresponds to the structure of hydrolyzed PENV subsequently decarboxylated (penilloic acid of PENV). The assignment of peaks in the MS spectrum that lead to this proposal is shown in (A, B) (Supplementary Data). It was not possible to register the MS spectrum of **MET03** given its low intensity. However, the *m*/*z* value determined for this compound is in good agreement with that of the glucuronidated derivative of **MET02**. This kind of derivative has been described for other β-lactam antibiotics such as PENG.^15^ The structure of **TP13** was proposed with the help of MassFrontier software. This compound can be formed after decomposition of the thiazolidine ring from either **MET02** or **PENV-HYDRO**.

### 3.6 Proposed Biomarker Candidates for the Pharmacological Treatment

As described in Section 3.4 (Figure 3), only **PENV-HYDRO**, **MET02,** and **MET03** are detectable in samples after some cooking treatments. **MET02** is detectable not only in DT samples but also in PT samples, while **MET03** and **PENV-HYDRO** are only detectable in DT samples using LC-LTQ-Orbitrap. Nevertheless, given its high sensitivity, LC-ESI-QqQ is usually used instead to analyze the presence of antibiotics in food from animal origin.

**Figure 4:**
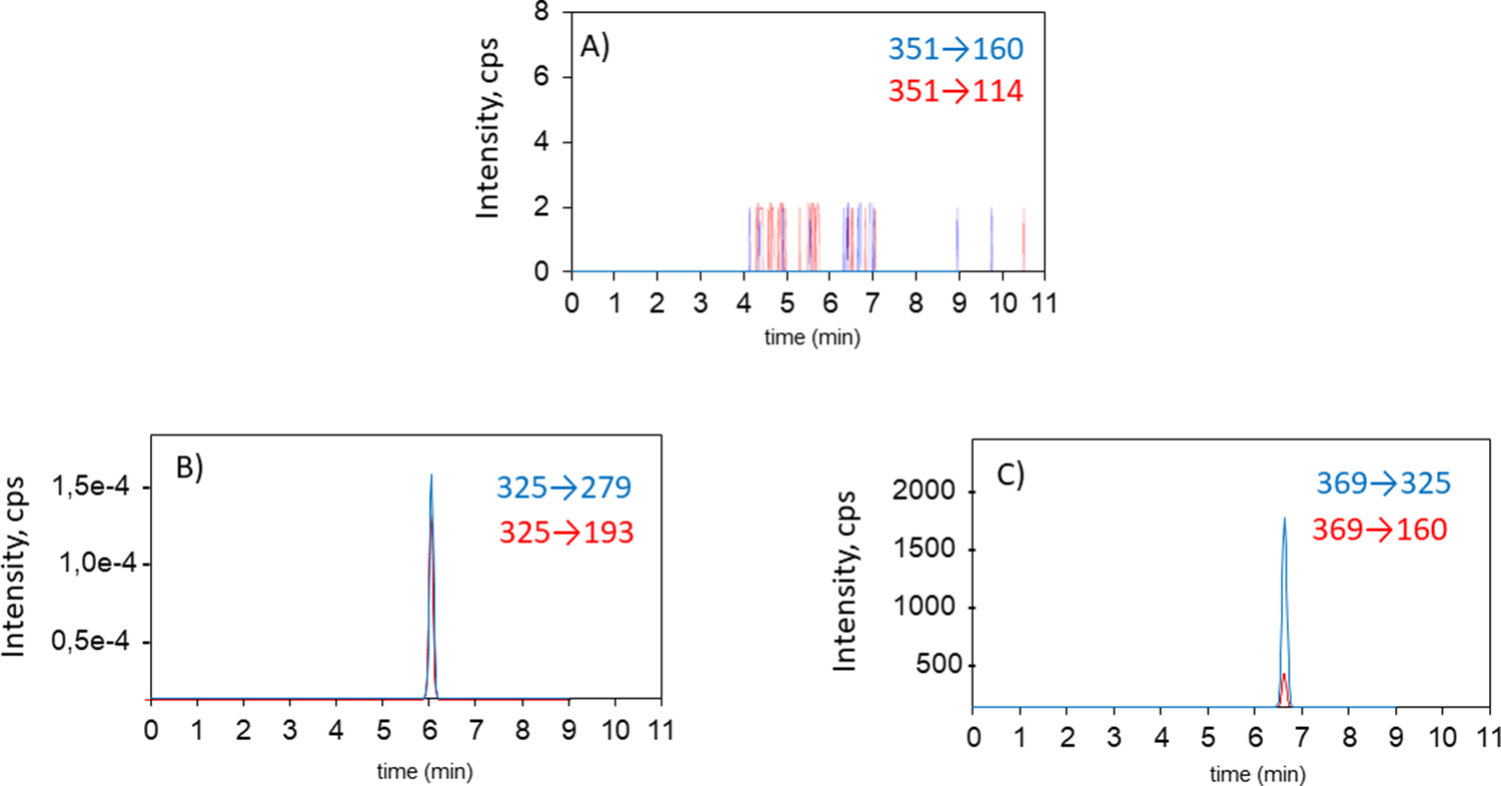
Chromatograms of the liver samples, after 5 days of pharmacological treatment was stopped, obtained by LC-ESI-QqQ. (A) Transitions 1 and 2 of the **PENV**. (B) Transitions 1 and 2 of **MET02**. (C) Transitions 1 and 2 of **PENV-HYDRO**.

To determine if any of the above-mentioned metabolites can become useful biomarkers for treatment with PENV, different transitions were monitored using LC-ESI-QqQ in samples from medicated animals. Results were compared with those obtained for PENV, the parent compound. In Figure 4, the chromatograms obtained for **BW** of the liver samples of animals slaughtered 5 days after stopping the pharmacological treatment (**5PT**) are shown. Given the similar behavior of **MET03** and **PENV-HYDRO** in these samples and the higher intensity of this later, **MET03** was discarded at this level. For PENV (Figure 4A), the two transitions (quantification transition (351 → 160 (15 V)) and identification transition (351 → 114 (45 V)) are depicted. Unfortunately, no signal for PENV was detected. This is in good agreement with the results shown in Table A (Supplementary Data). As it is there indicated, PENV was observed only in **3DT** and **5DT** in **R** samples and cannot be used to determine PENV in cooked samples. Figure 4B,C shows the chromatograms for **MET02** and **PENV-HYDRO** for the same samples. The transitions (325 → 279 (15 V)) and (325 → 193 (45 V)) are monitored for metabolite **MET02,** while the transitions (369 → 325 (25 V)) and (369 → 160 (45 V)) are monitored for **PENV-HYDRO**. **MET02** and **PENV-HYDRO** present the highest relative abundances considering that the samples analyzed correspond to 5 days after the treatment was stopped (**5PT**). These compounds show enough persistence in time and thermal stability to be considered suitable biomarker candidates for pharmacological treatment instead of the parent compound. Additionally, the peaks corresponding to **MET02** are significantly more intense than those for **PENV-HYDRO**. In conclusion, we propose the use of **MET02**, and the indicated transitions to quantify and identify this metabolite, to perform the control of samples coming from animals medicated with PENV.

## Supporting Information Available

Concentration of PENV in tissues, transformation products, and structural elucidation (PDF)
