# Supplementary material for: Search for Biomarkers for the LC-ESI-QqQ Determination of Phenoxymethylpenicillin Treatment in Raw or Cooked Chicken Meat Samples
Source: J Agric Food Chem. 2024 May 29;72(23):13393–401. doi: 10.1021/acs.jafc.4c02060 (PMC11181315; doi:10.1021/acs.jafc.4c02060)
Supplement: Supplementary file 1 — jf4c02060_si_001.pdf [file jf4c02060_si_001.pdf]

SUPPLEMENTARY DATA

SEARCH FOR BIOMARKERS FOR THE LC-ESI-QqQ DETERMINATION OF PHENOXYMETHYLPENICILLIN TREATMENT IN RAW OR COOKED CHICKEN MEAT SAMPLES

Javier Giménez-López[1], Jéssica Jiménez-Murcia[1], Alexandra Junza[2,3], Cristina Minguillón[1], Dolores Barrón*[1,4]

(1) Departament de Nutrició, Ciències de l'Alimentació i Gastronomia, Campus de l'Alimentació de Torribera, Universitat de Barcelona. Avda. Prat de la Riba 171, 08921 Sta Coloma de Gramenet, Barcelona, Spain.

(2) Department Enginyeria Química i Química Analítica, Universitat de Barcelona. Martí i Franquès, 1-11, 08028 Barcelona, Spain.

(3) Unitat d'Ànalisi Química. Espectrometria de Masses. Servei de Recursos Científics i Tècnics, Universitat Rovira i Virgili, Avinguda dels Països Catalans, 26. Campus Sescelades. 43007 Tarragona. Spain (Present Address)

(4) Institut de Recerca en Nutrició i Seguretat Alimentaria. Universitat de Barcelona, (INSA-UB). Institut de Recerca en Nutrició i Seguretat Alimentaria. Universitat de Barcelona (INSA-UB, Recognized as a Maria de Maeztu Unit of Excellence Grant (CEX2021-001234-M)).

**To whom correspondence should be addressed.*

Phone: +34934020917 e-mail: dolores.barron@ub.edu

ASSOCIATED CONTENT

TABLE A. Quantification of PENV in muscle and liver of medicated chicken.

TABLE B. Transformation products found in contact with matrices and in pH.

FIGURE A. Structural elucidation of **MET02** by LC-LTQ-Orbitrap.

FIGURE B. Structural elucidation of **PENV-HYDRO** by LC-LTQ-Orbitrap.

Table A. Quantification of PENV in muscle and liver of medicated chicken.

		Muscle		Liver	
	Treatment status*	Animal	Concentration (SD) µg·kg^{-1}	Animal	Concentration (SD) µg·kg^{-1}
**RAW samples**	3DT	A1	7.2 (0.3)	A1	17 (3)
	3DT	A2	10.1 (0.8)	A2	< LOD
	5DT	A3	17 (2)	A3	< LOD
	5DT	A4	14 (1)	A4	68 (15)
	3PT	A5	< LOD	A5	< LOD
	3PT	A6	< LOD	A6	< LOD
	5PT	A7	< LOD	A7	< LOD
	5PT	A8	< LOD	A8	< LOD
**GRILLING samples**	3DT	A1, A2	< LOQ	A1	< LOQ
	5DT	A3, A4	< LOQ	A4	< LOQ
	3PT	A5, A6	< LOD	A5	< LOD
	5PT	A7, A8	< LOD	A7	< LOD
**BOILING samples**	3DT	A1, A2	< LOQ	A1	< LOD
	5DT	A3, A4	< LOQ	A4	< LOD
	3PT	A5, A6	< LOD	A5	< LOD
	5PT	A7, A8	< LOD	A7	< LOD
**BOILING WATER samples**	3DT	A1, A2	< LOD	A1	< LOD
	5DT	A3, A4	< LOD	A4	< LOD
	3PT	A5, A6	< LOD	A5	< LOD
	5PT	A7, A8	< LOD	A7	< LOD

*XDT: days during treatment; XPT: days post-treatment

Table B. Transformation products found in contact with matrices and in pH

m/z		Formula	RDB	Error (ppm)	Conditions in which ion is detected	Matrix	ACRONIM
151.0241		$C_5H_6O_3NNa^+$	3	-0.73	Contact with matrix	L	**TP01**
151.1481		$C_{11}H_{19}^+$	3	0	Contact with matrix	L	**TP02**
156.0818		$C_6H_{15}NSNa^+$	0	-0.6	Contact with matrix	M	**TP03**
164.1080		Undetermined	-	-	Contact with matrix	M	**TP04**
168.1029		Undetermined	-	-	Contact with matrix	M	**TP05**
174.0759		$C_7H_{12}NO_4^+$	3	1.1	Contact with matrix	L	**TP06**
242.0729		$C_{10}H_{14}O_3N_2S^+$	5	-3.7	Contact with matrix	M	**TP07**
249.1484		$C_{15}H_{21}O_3^+$	6	0.4	Contact with matrix	L	**TP08**
320.0469		$C_{15}H_{11}N_3O_2SNa^+$	12	-1.6	Contact with matrix	L	**TP09**
325.1238		$C_{15}H_{21}N_2O_4S^+$	7	6.5	Contact with matrix	M	**TP10**
327.0205		Undetermined	-	-	Contact with matrix	L	**TP11**
351.1014		$C_{16}H_{19}N_2O_5S^+$	9	-1.4	Parent compound	M, L	**PENV**
351.1014		$C_{16}H_{19}N_2O_5S^+$	9	-1.4	pH 2.0	--	**PENV-DIKETO**
369.1118		$C_{16}H_{21}N_2O_6S^+$	8	-0.8	Contact with matrix, pH 2, 4, 8	M, L	**PEN-HYDRO**
373.0856		$C_{18}H_{17}N_2O_5S^+$	12	-0.8	Contact with matrix	M	**TP12**

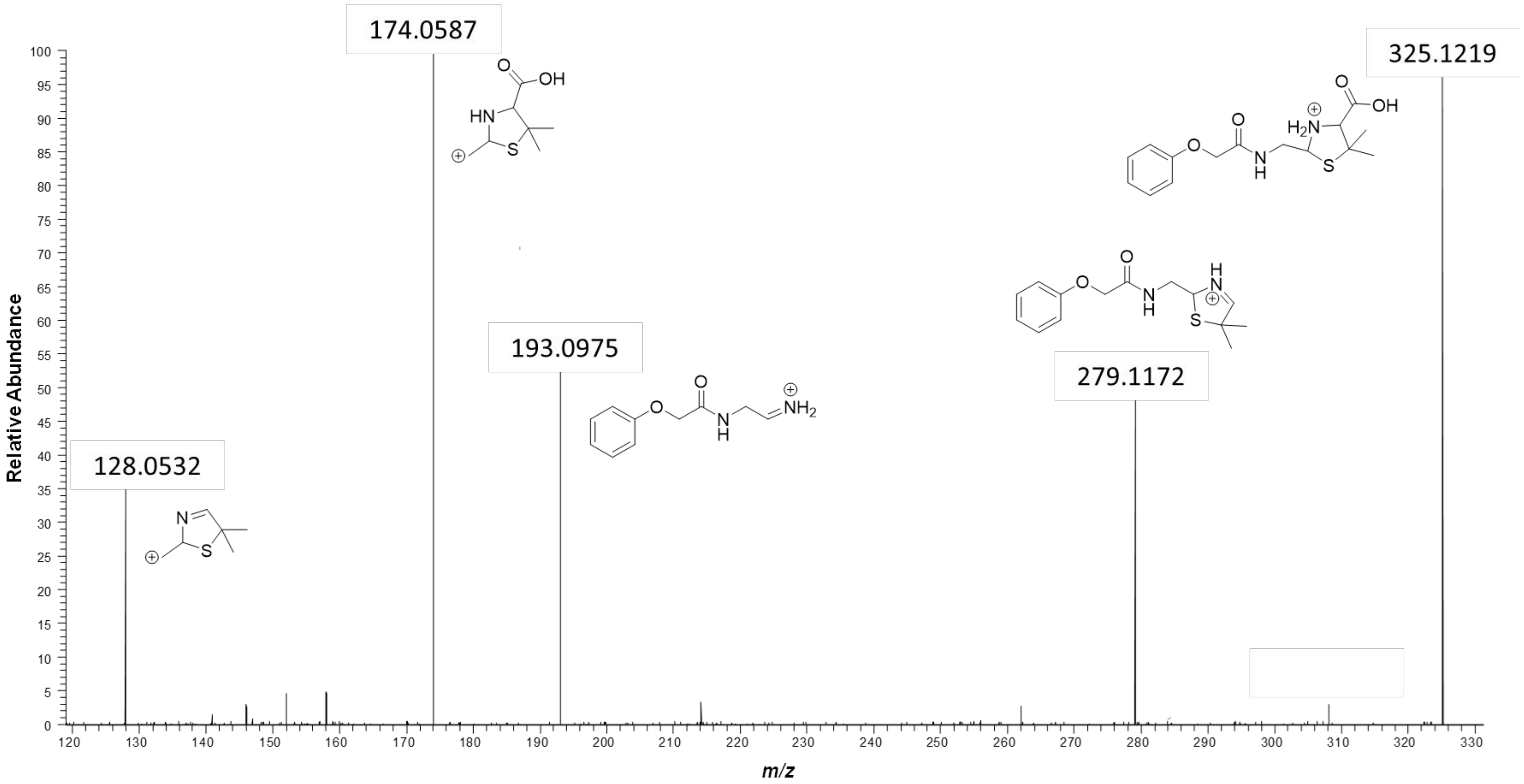

Figure A

Structural elucidation of **MET02** by LC-LTQ-Orbitrap.

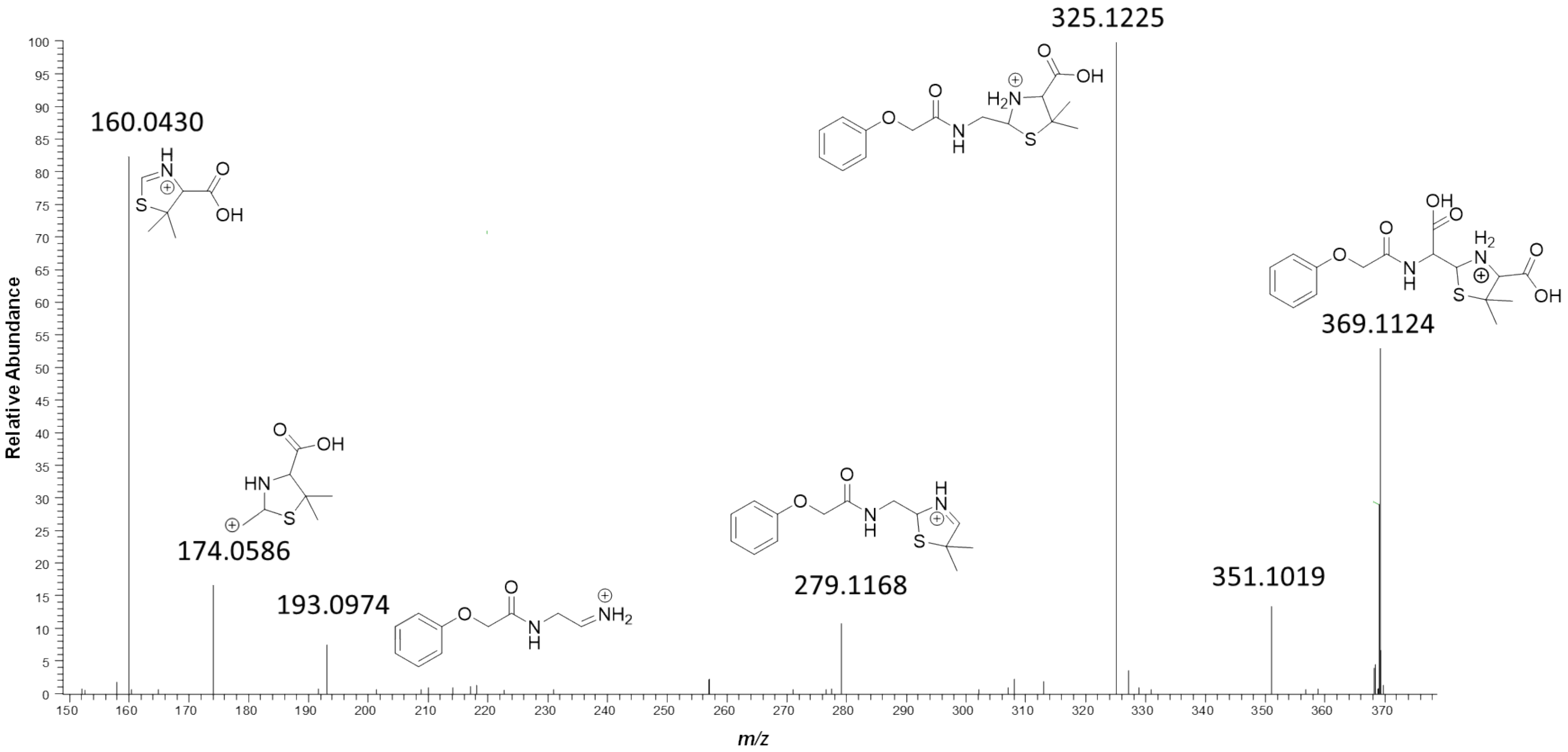

Figure B

Structural elucidation of **PENV-HYDRO** by LC-LTQ-Orbitrap.
